# Improved cardiac gating at 3T with the “3D-QRS” method utilizing MRI-compatible 12-lead ECGs

**DOI:** 10.1186/1532-429X-15-S1-W20

**Published:** 2013-01-30

**Authors:** Z Tse, C Dumoulin, R Watkins, K Butts Pauly, RY Kwong, GF Michaud, W Stevenson, F Jolesz, EJ Schmidt

**Affiliations:** 1 Engineering, The University of Georgia, Athens, GA, USA; 2 Radiology, Cincinnati Children’s Hospital Medical Center, Cincinnati, OH, USA; 3 Radiology, Stanford University, Stanford, CA, USA; 4 Cardiology, Brigham and Women’s Hospital, Boston, MA, USA; 5 Radiology, Brigham and Women’s Hospital, Boston, MA, USA

## Background

Blood is rapidly ejected from the left ventricle during early systole, travels in the aortic arch perpendicular to the MRI’s main field, thus generating a Magnetohydrodynamic (MHD) voltage which is larger than the real ECG QRS complex in high field MRI. The MHD voltage (VMHD) is severally irregular in patients with arrhythmia, since arrhythmic ECG beats are interleaved between successive sinus rhythm (SR) beats. The VMHD overlay in ECG traces can result in intermittent QRS detection, leading to blurred images and longer scan times. Since accurate gating is essential for successful cardiac imaging, we developed a “3D-QRS” method for real-time detection of the QRS complex based on 12-lead ECG traces acquired inside the MRI. We validated this method at 3T in patients with Premature Ventricular Contractions (PVCs), Atrial Fibrillation (AF) and in an exercising athlete with time-varying heart rate.

## Methods

**Figure 1:**
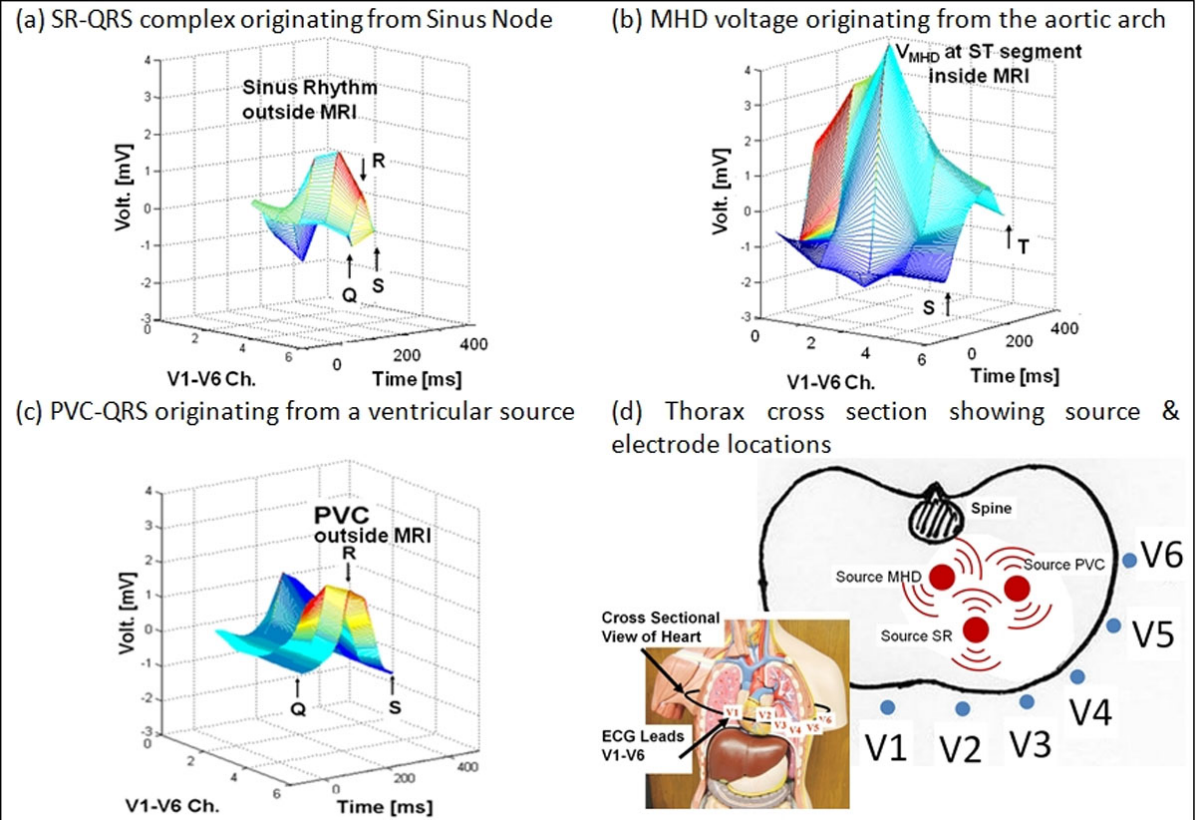
3-D representations of the (a) SR-QRS complex, (b) V_MHD_ and (c) the PVC-QRS complex, all of which have unique 3-D geometrical kernels. (d) Electrical signal propogate via different paths from the various heart sources to the surface leads V1-V6, which is reflected in their ECG channel's axes.

An MRI-compatible ECG system [1] was constructed and tested with a GE 3T MRI. It acquired 12-lead ECG traces, computed the “3D-QRS” algorithm for QRS detection, and sent out gating triggers to the scanner gating unit. The 3D-QRS was based on a new representation of the ECG channels, where ECG channels V1-V6 became the third spatial axis, adding a dimension to the conventional ECG time and voltage axes. Fig. 1(a-b) shows the 3D representation of the SR-QRS complex and VMHD. As the QRS complex and VMHD originate from the sinus node and the aortic arch respectively, the channel axis carried information on the propagation from the different cardiac sources (SR, MHD, PVC) to the surface leads (Fig. 1(d)). 3D-QRS presented a unique 3-D kernel of the QRS complex which could be clearly differentiated from VMHD even at 3T. 3D-QRS kernels were also used for separating SR from PVC beats using similar principles (Fig. 1(a,c)). A FFT 2D-cross-correlation subroutine was then used in Matlab to achieve a computational speed of <30msec for detecting QRS complexes in real-time.

## Results

**Figure 2:**
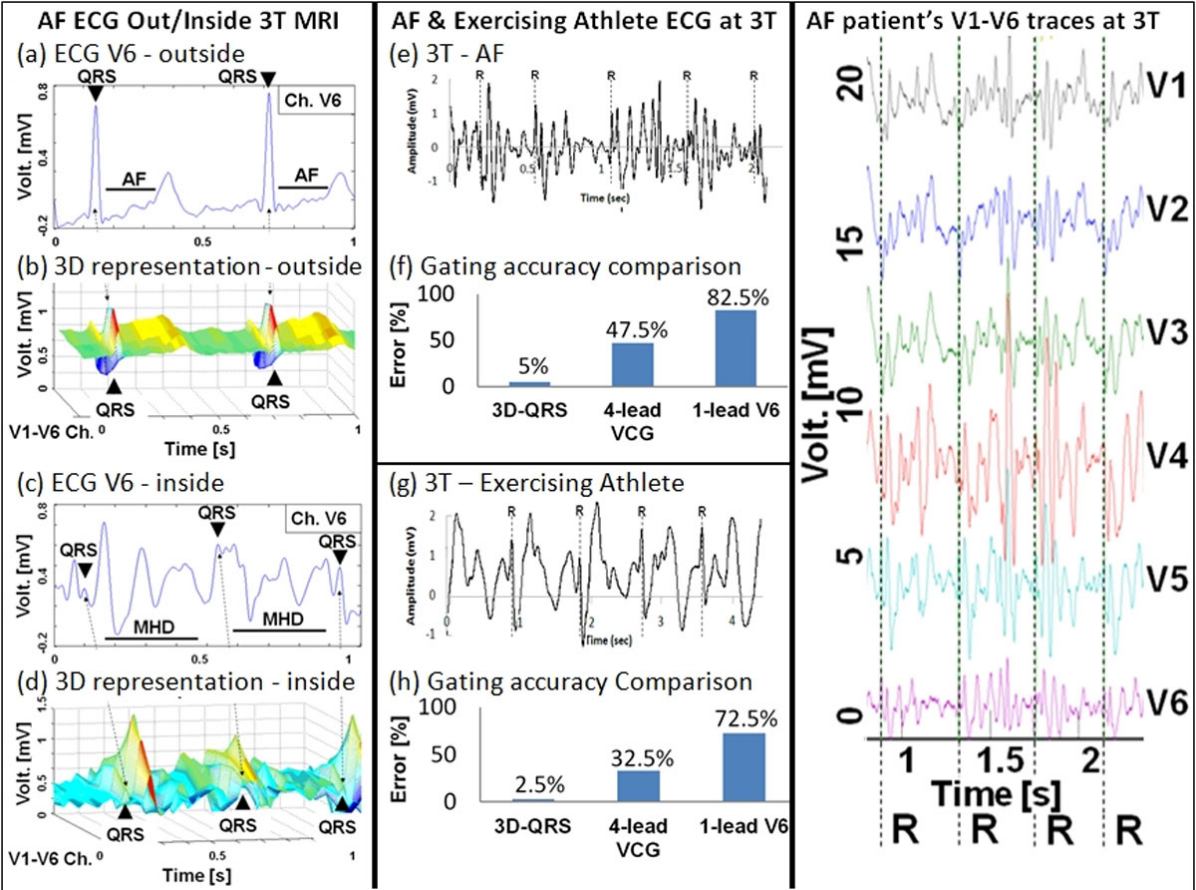
QRS detections using the 3D-QRS method in 3T MRI in patients with AF, and in an exercising athlete. "R" designates detected R-wave peaks using the 3D-QRS algorithm.

Fig. 2(a-d) shows ECGs of an AF patient taken out/inside a 3T MRI: the QRS complexes in V6 at Fig.2(c) are less than 10% of the VMHD amplitude and failed to be identified with conventional 4-lead ECG gating, while the SR-QRS kernels at Fig.2(d) were distinguishable based on their unique 3-D features. Fig. 2(e-h) shows the gating results achieved in 2 AF patients and an exercising athlete subject which produced heart rate variations from 44-87bpm. Gating accuracy was compared in Fig.2(f,h) between 3D-QRS, 4-lead Vectorcardiogram (VCG) and 1-lead Pan-Tompkins algorithm based on V6. 2 set of 20-sec breath-held ECGs were considered in all cases.

## Conclusions

In high-field MRI, the 3D-QRS method allowed accurate detection of the QRS, and beat-type separation in arrhythmia patients, which allowed for real-time scanner gating.

## Funding

NIH U41-RR019703, R43 HL110427-01, AHA 10SDG261039
